# Cross Talk Between Macrophages and Podocytes in Diabetic Nephropathy: Potential Mechanisms and Novel Therapeutics

**DOI:** 10.1155/mi/8140479

**Published:** 2025-05-02

**Authors:** Siming Yu, Zehui Han, Chunsheng Li, Xinxin Lu, Yue Li, Xingxing Yuan, Dandan Guo

**Affiliations:** ^1^Department of Nephrology II, First Affiliated Hospital of Heilongjiang University of Chinese Medicine, Harbin 150036, China; ^2^First Clinical Medical College, Heilongjiang University of Chinese Medicine, Harbin 150040, China; ^3^Department of Gastroenterology, Heilongjiang Academy of Traditional Chinese Medicine, Harbin 150006, China; ^4^Department of Cardiology, Second Affiliated Hospital of Heilongjiang University of Chinese Medicine, Harbin 150001, China

**Keywords:** apoptosis, autophagy, diabetic nephropathy, macrophage, podocyte, pyroptosis

## Abstract

Diabetic nephropathy (DN) is a leading cause of chronic kidney disease and end-stage renal failure worldwide. Podocytes, essential components of the glomerular filtration barrier (GFB), are profoundly affected in the diabetic milieu, resulting in structural and functional alterations. Concurrently, macrophages, pivotal innate immune cells, infiltrate the diabetic kidney and exhibit diverse activation states influenced by the local environment, playing a crucial role in kidney physiology and pathology. This review synthesizes current insights into how the dynamic cross talk between these two cell types contributes to the progression of DN, exploring the molecular and cellular mechanisms underlying this interaction, with a particular focus on how macrophages influence podocyte survival through various forms of cell death, including apoptosis, pyroptosis, and autophagy. The review also discusses the potential of targeting macrophages to develop more effective treatments for DN.

## 1. Introduction

Diabetic nephropathy (DN) is a primary factor in the development of end-stage renal disease, represents a significant public health challenge, affecting millions worldwide [1]. The pathophysiology of DN is complex, involving a myriad of cellular and molecular pathways. The kidney's glomerulus is essential for the filtration of blood, a process crucial for maintaining homeostasis. Podocytes, with their unique structure and function, are involved in the formation of the glomerular filtration barrier (GFB) [2]. In DN, hyperglycemia and other metabolic disturbances initiate a cascade of events that lead to podocyte injury, characterized by effacement, detachment, and loss of these cells. Moreover, glucose-induced damage to other renal cells, including mesangial cells, endothelial cells, and tubular epithelial cells, results in mesangial expansion, glomerulosclerosis, and tubulointerstitial fibrosis [3, 4]. These pathological changes disrupt the GFB, leading to proteinuria, a hallmark of DN [5].

Macrophages, multifunctional cells of the innate immune system, are known for their dual roles in both promoting and resolving inflammation [6]. In the diabetic milieu, macrophages infiltrate the kidney and become activated, assuming a spectrum of activation states influenced by the local microenvironment [7]. Tissue-resident macrophages exert various functions in diabetic kidney injuries by secreting cytokines, recruiting peripheral monocytes, and exacerbating renal damage [8]. The cross talk between macrophages and podocytes in DN is mediated through a variety of mechanisms [9, 10]. Under diabetic stress, podocytes release cytokines and chemokines that attract and activate macrophages [11, 12]. Conversely, activated macrophages secrete a range of factors that sculpture podocyte function and further affect their survival by regulating various forms of podocyte death, such as apoptosis, pyroptosis, and autophagy [13–15]. The identification of molecular mechanisms involved in the macrophage–podocyte cross talk opens new avenues for therapeutic intervention.

This review aims to elucidate the current understanding of macrophages and podocytes in renal physiology and DN, highlighting the latest research findings and their implications for the interaction between macrophages and podocytes, as well as for the development of promising therapeutic strategies in this disease.

## 2. Podocytes in DN

Podocytes are highly specialized glomerular visceral epithelial cells with a unique morphology characterized by a cell body primary processes and interdigitating foot processes (FPs) [16]. The FPs envelop the glomerular capillaries and are connected by the slit diaphragm (SD), a specialized junctional complex essential for filtration. SD proteins consist of nephrin, podocin, and P-cadherin, which form a porous structure allowing selective filtration of blood while preventing protein leakage [17]. The cytoskeleton of podocytes, predominantly composed of actin filaments, undergoes dynamic remodeling in response to various stimuli, ensuring the adaptability and integrity of the GFB [18]. In addition to preventing proteinuria by maintaining the GFB, podocytes secrete various growth factors and cytokines that regulate glomerular function and response to injury [19].

As a hallmark of DN, the loss of podocytes is closely related to the severity of proteinuria and the progression of glomerulosclerosis [20]. At the early stage of DN, glomerular hypertrophy and hyperfiltration occur, imposing additional mechanical stress on podocytes, which leads to podocyte detachment from the glomerular basement membrane and persistent cell damage [21]. Injured podocytes exhibit FP effacement, a morphological alteration reflected by flattening, widening, and retraction of the normally interdigitating FPs, which disrupts the expression and localization of SD proteins, such as nephrin and podocin, impairing of the filtration function of podocytes and causing the leakage of proteins into the urine [22, 23]. Within podocytes, unfolded protein responses and reactive oxygen species (ROS) overproduction are induced under the hyperglycemic environment of diabetes, which further triggers endoplasmic reticulum stress and oxidative stress, causing damage to podocyte DNA, proteins, and lipids, ultimately leading to cellular dysfunction and death [5, 24]. With DN progression, amounts of inflammatory cytokines, like tumor necrosis factor (TNF)-*α* and interleukin (IL)-1*β*, are produced in the diabetic milieu, which harm podocytes and disrupt their functions [25]. In response to hyperglycemia, impaired autophagy in podocytes triggers the aggregation of damaged organelles and proteins, which exacerbates cell injury; moreover, several cell death processes, including apoptosis, pyroptosis, necroptosis, and ferroptosis, are enhanced in podocytes, which are regarded as the major contributors for podocyte loss in DN [26, 27].

Therefore, podocyte loss is critically involved in the pathogenesis of DN, undergoing a range of structural and functional changes. Elucidating these pathologic alterations is essential for developing targeted therapies to preserve podocyte function and prevent the progression of DN. In this regard, mitigating podocyte loss in DN represents promising therapeutic strategies for this disease. Presently, multiple cell death modes have been confirmed in podocytes, including apoptosis, pyroptosis, autophagy, necroptosis, and ferroptosis, each contributing to the pathogenesis of DN [27]. Podocyte apoptosis and resulting podocyte loss affect the early stages of diabetic kidney and contribute to diabetic glomerulopathy in both type I and type II diabetes [28]. Pyroptosis is also associated with the loss of podocytes and the glomerular injury in DN [29]. Similarly, high blood glucose levels are believed to impair autophagy, eliciting podocyte dysfunction and GFB damage [30]. Given the high sensitivity of podocytes to ROS, an overproduction of ROS causes irreversible alterations in the structure and function of these cells, leading to necroptosis and ferroptosis, ultimately to the development of DN [31, 32]. Injured podocytes release substantial amounts of proinflammatory cytokines, which recruit monocyte into the renal tissue where they are converted into macrophages that acquire the M1 phenotype under the inflammatory milieu and produce proinflammatory mediators, promoting renal damage and DN progression [6].

## 3. Macrophages in DN

### 3.1. Origins and Phenotypes of Macrophages

Macrophages are versatile and dynamic immune cells that exert a crucial function in kidney homeostasis and pathology. These cells reside as stationary cells within tissues from the yolk sac and fetal liver during embryonic development or originating from the differentiation of blood monocytes [33]. Embryonically derived macrophages represent a population of resident kidney macrophages and colonize the renal tissue during its development and continue to proliferate in situ throughout adulthood [34]. Recent study revealed that yolk–sac-derived macrophages gradually increase in number with age and become a significant component of the renal macrophage population in older mice [35]. Postnatally, the kidney macrophage pool is maintained and supplemented by circulating monocytes, which originate from hematopoietic stem cells located in the bone marrow [36]. These monocytes can infiltrate the kidney, especially during inflammation or injury, and differentiate into macrophages [37]. Renal macrophages, therefore, are sustained through both local proliferation and recruitment from circulating progenitors. They exhibit a range of phenotypes and functions that are influenced by the kidney's microenvironment, playing critical roles in tissue homeostasis, injury response, and repair [38]. Thus, these macrophages possess high plasticity to adapt in different microenvironments.

**Figure 1:**
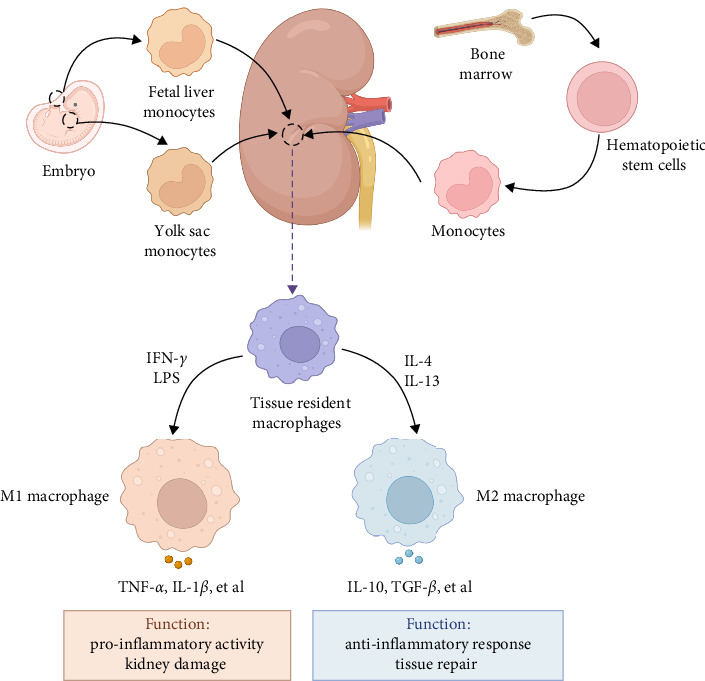
Origins and phenotypes of macrophages in kidney. Renal macrophages can be originated from the differentiation of blood monocytes derived from hematopoietic stem cells in the bone marrow or exist as resident cells in tissues from early embryonic development. Tissue resident macrophages can be induced to form the M1 phenotype by LPS or IFN-*γ* stimulation and secrete inflammatory mediators, such as TNF-*α* and IL-1*β*, to exert a pro-inflammatory effect; moreover, they can also be transformed into the M2 phenotype activated by IL-4 and IL-13 and promote tissue repair via producing anti-inflammatory cytokines like IL-10 and TGF-*β*. This macrophage phenotype switch is a reversible and dynamic process in a diseased microenvironment in the DN. IFN, interferon; IL, interleukin; LPS, lipopolysaccharide; TGF, transforming growth factor; TNF, tumor necrosis factor.

The traditional dichotomy of M1 (pro-inflammatory) and M2 (anti-inflammatory or reparative) macrophages is an oversimplification, but provides a framework for understanding macrophage function [39]. M1 macrophages, typically induced by interferon (IFN)-*γ* and lipopolysaccharide (LPS), secrete cytokines like inducible nitric oxide synthase (iNOS), TNF-*α*, and IL-1*β*, which trigger pro-inflammatory responses and are associated with kidney injury and fibrosis [40]. In the initial phase of kidney injury, macrophages are stimulated by pathogen-associated molecular patterns, danger-associated molecular patterns, and pro-inflammatory cytokines, differentiating into pro-inflammatory M1 macrophages that aggravate tissue damage [41, 42]. Persistent M1 macrophages activation and resultant inflammatory damage cause a reduced renal function and ultimately fibrosis [43]. M2 macrophages, often induced by IL-4 and IL-13, express markers, such as arginase (Arg)1, CD206, and IL-10, are responsible for inflammation resolution, tissue repair, and fibrosis [44]. Besides, factors such as cytokines, growth factors, and metabolic cues in the renal microenvironment significantly influence macrophage phenotype and function [45]. For example, in response to kidney injury, macrophages transition from pro-inflammatory M1 to a pro-reparative M2 phenotype characterized by expression of Arg1, which is required for the tubular cell proliferation that mediates renal repair [46]. Therefore, macrophages exist along a spectrum of activation states, influenced by the local microenvironment, allowing them to adapt to various physiological and pathological conditions (Figure 1). Of interest, M2 macrophages could be further classified as M2a, M2b, M2c, and M2d subcategories, each characterized by unique cell surface markers, secreted cytokines, and biological functions [47]. However, the role of these M2 macrophage subtypes in DN remains unclear.

### 3.2. Pathogenic Roles of Macrophages in DN

During DN progression, infiltrating macrophages have emerged in human diabetic kidneys and are linked to the deteriorating renal function observed in patients with DN [9]. It is reported that macrophages are found in both the glomeruli and interstitium of type 2 diabetic patients with DN, with a 2:1 ratio of M1 to M2 types, which are related to interstitial fibrosis, tubular atrophy, glomerulosclerosis, and albuminuria [48]. Further evaluation of the RNA sequencing data of patients with advanced DN indicates a significant increase in macrophage numbers compared to those in patients with early DN and the control [49]. At the early stage of DN, M1 macrophages are recruited into the kidney, while M2 macrophages predominate during the late stage, as evidenced by the M1/M2 ratio peaks at the early stage of DN and subsequent drops in the late stage of DN [50]. These findings suggest that initial M1 macrophages infiltration in the kidney of DN exert pro-inflammatory function and promotes kidney injury; but with disease progression to the late stage, these macrophages locally differentiate to M2 macrophages, which mediate tissue repair and renal fibrosis. Indeed, the transition between M1 and M2 macrophages is regarded as a dynamic process depending on the renal microenvironment during DN progression [51]. Moreover, increased macrophage numbers are accompanied with frequency changes in regulatory T (Treg) cells and T helper 2 (Th2) cells in diabetic kidney tissues [52], indicating that macrophages might influence other immune cells in the kidney microenvironment. Further clarifying the role of various immune components and their cross talk with macrophages are important for understanding the pathogenesis of DN.

It is believed that macrophages exhibit a dynamic range of phenotypes in DN, transitioning from pro-inflammatory M1 to anti-inflammatory M2 [51]. Initially, hyperglycemia is a key driver for the recruitment of macrophages to the kidney, as high glucose (HG) levels stimulate the production of chemokines and adhesion molecules in renal cells, facilitating the migration of macrophages into the renal tissue [53]. Advanced glycation end products (AGEs), formed in the diabetic milieu, subsequently activate macrophages through receptors for AGE, which triggers the release of pro-inflammatory cytokines, such as TNF-*α*, IL-1*β*, and IL-6, as well as chemokines like monocyte chemotactic protein-1 (MCP-1), contributing to the inflammatory milieu in the kidney [54]. Besides, these pro-inflammatory macrophages produce amounts of ROS and directly cause damage to renal cells and exacerbate inflammation [55]. They also interact with other immune cells, such as T cells and dendritic cells, amplifying the inflammatory response and oxidative stress, leading to glomerular and tubulointerstitial damage [10]. At the later stage after renal injury, various cytokines are generated in the tissue environment, such as IL-4, IL-13, and transforming growth factor (TGF)-*β*, which are responsible for the activation of M2 macrophages [6]. Hence, there is a shift towards M2 macrophages, which are involved in tissue repair and fibrosis. In response to renal damage, these macrophages secrete cytokines like TGF-*β* to stimulate the proliferation of myofibroblasts and the accumulation of extracellular matrix components, participating in the repairing process [56]. However, excessive release of fibrotic factors like TGF-*β* by macrophages induce the epithelial-to-mesenchymal transition (EMT) in tubular epithelial cells, leading to glomerulosclerosis and tubulointerstitial fibrosis [6]. Thus, macrophages represent significant plasticity in DN, with their phenotype influenced by the local microenvironment and disease stage, contributing to both renal injury and fibrosis [9]. Therapeutic strategies aimed at modulating macrophage phenotypic transformation might be promising in slowing the progression of DN.

## 4. Cross Talk Between Podocytes and Macrophages in DN

### 4.1. Effects of Podocytes on Macrophage Activation

As a critical event in DN, podocyte damage precedes and triggers macrophage recruitment by releasing factors that attract macrophages to the site of injury [57]. For example, in response to hyperglycemic stress, damaged podocytes produce chemokines such as MCP-1, potent recruiters, and activators of macrophages [58, 59]. An increased expression of vascular endothelial growth factor (VEGF) in podocytes is correlated to infiltration of macrophages in injured glomeruli of diabetic mice, suggesting that VEGF could serve as a chemotactic factor for macrophage migration [12]. In human podocytes treated with growth hormone (GH), the TNF-*α* signaling is elevated to promote the differentiation of monocytes into macrophages, while inhibiting either GH activity or TNF-*α* expression in podocytes reduces macrophage recruitment, glomerular injury, and proteinuria [11]. In addition, factors produced by injured podocytes influence the phenotype of macrophages. For instance, podocyte-derived pro-inflammatory cytokines skew macrophages towards the M1 phenotype, exacerbating glomerular inflammation and injury in DN [36]. By the interaction of podocyte-derived ligands with macrophage Toll-like receptors (TLRs), various inflammatory pathways like the nuclear factor (NF)-*κ*B signaling are activated in macrophages, leading to the production of pro-inflammatory cytokines and chemokines and ultimately to the inflammatory milieu in DN [60].

### 4.2. Effects of Macrophages on Cell Death Pathways of Podocytes

#### 4.2.1. Apoptosis

As a type of programmed cell death, apoptosis can be stimulated by the death receptor pathway and the mitochondrial pathway [61]. During DN progression, chronic hyperglycemia acts as a prominent inducer of podocyte apoptosis. HG leads to the accumulation of AGEs, which bind to receptors on podocytes, triggering apoptotic pathways. For example, the interaction between AGEs and CXC chemokine ligand (CXCL) 9 activates the JAK2/STAT3 pathway in podocytes, further causes reduced levels of Bax/Bcl-2 and activated caspase-3, suggesting that AGEs exert proapoptotic effects in podocytes [62]. Accumulation of AGEs promotes overproduction of ROS and activation of protein kinase C (PKC), both of which mediate podocyte apoptosis [63]. ROS-induced oxidative stress further exacerbates podocyte apoptosis and subsequent loss of podocytes, which contribute to glomerular damage and progression of DN [64].

Macrophages are confirmed to affect podocyte apoptosis. In vitro assays of macrophages and podocytes found that HG induces M1 macrophage phenotype and podocyte apoptosis in a dose-dependent manner and reduces sirtuin 6 (SIRT6) expression; moreover, overexpressing SIRT6 in macrophages activates M2 transformation and protects the podocytes from HG-induced apoptosis, as evidenced by the increased expression levels of Bcl-2 and CD206, as well as by the downregulation of the expression levels of Bax and CD86 [13]. SIRT6 inhibits mitochondrial dysfunction and exerts antiapoptotic effects via stimulating the adenosine monophosphate-activated protein kinase (AMPK) pathway in HG-stimulated podocytes [65]. In fact, AMPK activation is required for the suppression of oxidative stress-mediated apoptosis of podocytes [66]. However, whether macrophages regulate the expression of SIRT6 in podocytes and further affect podocyte apoptosis is elusive. Compared with control rats, podocyte apoptosis increases in STZ-induced diabetic rats, which is related to increased macrophages infiltration in the kidney. Further mechanistic investigations explained that HG-activated M1 macrophages secrete TNF-*α*, which induces excess ROS generation in podocytes and causes cell apoptosis via activating the p38 mitogen-activated protein kinase (MAPK) pathway [67]. This signaling pathway further activates NF-*κ*B, which increases pro-inflammatory cytokine expression and cell apoptosis in podocytes and renal tissues [68]. Thus, these findings suggest that macrophages can regulate podocyte apoptosis through affecting HG-induced mitochondrial dysfunction and oxidative damage.

#### 4.2.2. Pyroptosis

Unlike apoptosis, pyroptosis is a pro-inflammatory form of cell death, which has significant implications for kidney inflammation and injury in DN. Inflammasomes, particularly the PYD domains-containing protein 3 (NLRP3) inflammasome, play a crucial role in initiating podocyte pyroptosis. Hyperglycemia and oxidative stress in the diabetic milieu activate NLRP3, facilitating the cleavage of pro-caspase-1 into active caspase-1, which then promotes the maturation and secretion of key pro-inflammatory cytokines, IL-1*β* and IL-18 [69]. Caspase-1 activation also induces the cleavage of gasdermin D (GSDMD). The N-terminal fragment of GSDMD creates pores in the cell membrane, resulting in cell swelling, membrane rupture, and release of inflammatory contents, characteristic of pyroptosis [70]. The cumulative effect of podocyte loss, inflammation, and fibrosis due to pyroptosis contributes to the progression of DN [71].

It has been demonstrated that extracellular vesicles derived from macrophages can trigger cell pyroptosis by activating the NLRP3 inflammasome [72]. Indeed, in HG-stimulated macrophage-derived extracellular vesicles, the level of miR-21-5p is upregulated, which facilitates the production of ROS and activation of inflammatory response in podocytes. Molecular investigation verified that miR-21-5p suppresses the A20 expression, which induces ROS generation and inflammasome activation, provoking podocyte pyroptosis [14]. As a crucial activator of NLRP3, A20 is verified to promote cellular damage and tissue inflammation [73]. Therefore, these results indicate that macrophages can affect podocyte pyroptosis during DN progression.

#### 4.2.3. Autophagy

Autophagy, a cellular process for degrading and recycling cellular components, plays a significant role in podocyte function, particularly in the context of DN. Under diabetic conditions, activated autophagy is initially protective in podocytes, aiming to remove damaged proteins and organelles and mitigate stress-induced damage [74]. However, chronic hyperglycemia can lead to dysregulated autophagy in podocytes and is characterized by either excessive or insufficient autophagic activity, both of which can contribute to podocyte injury and promote the progression of DN [75]. Several signaling pathways, such as mTOR and AMPK, as well as autophagy-related genes (ATGs) like ATG5 and ATG7, are essential for the regulation of autophagic process in podocytes [76].

It is reported that macrophage-derived exosomes contain some gene regulators, like miRNAs, which can regulate ATGs and signaling pathways, and thus, affect autophagy in renal cell, such as mesangial cells and tubular epithelial cells, thereby participating in the development of DN [77, 78]. Consistent with these findings, overexpressed miR-25-3p is observed in M2 macrophage-derived exosomes that effectively ameliorates HG-induced podocytes injury. Further study revealed that miR-25-3p mitigates podocytes injury via triggering podocyte autophagy through suppressing the dual specificity protein phosphatase 1 (DUSP1) expression [15]. Inactivation of DUSP1 causes the increased expression of lipidated microtubule-associated protein light-chain 3 (LC3-II), an autophagy-associated protein, thus, activating cell autophagy [79]. Of interest, dysregulated autophagy in podocytes can trigger apoptosis, leading to podocyte loss, indicating that the balance between autophagy and apoptosis in podocytes is critical for cell survival [80]. Thus, whether macrophages simultaneously regulate autophagy and apoptosis of podocytes during DN progression merit further investigation.

#### 4.2.4. Necroptosis

Necroptosis is characterized by rupture of the cell membrane and the release of intracellular contents, leading to inflammation. This process is primarily mediated through the receptor-interacting protein kinase 1 and 3 (RIPK1/3) and the mixed lineage kinase domain-like protein [81]. In podocytes exposed to HG, activation of these pathways causes membrane disruption and cell death and ultimately leading to the breakdown of the GFB [82, 83]. In the context of DN, hyperglycemia and the resultant oxidative stress are key triggers for necroptosis, which stimulate the necroptosis pathway in podocytes, resulting in podocyte loss [84]. Besides, inflammatory cytokines, like TNF-*α*, are elevated in DN and participates in the activation of the RIPK1/RIPK3 signaling pathways, thereby potentiating necroptosis in podocytes [85]. Thereby, podocyte necroptosis plays a significant role in the pathogenesis of DN.

A recent study has demonstrated that the causality between macrophage activation and cell necroptosis. It is found that activated Notch1 in macrophage controls the RIPK3-mediated cell necroptosis through activation of *β*-catenin, suggesting that macrophages are involved in the regulation of necroptosis in surrounding cells [86]. The Notch1 signaling pathway has been demonstrated to affect the differentiation and necroptosis of various cell types [87]. A similar study unveiled that damaged renal tubular epithelial cells in DN recruit macrophages to the site of injury, where the Notch pathway activation in macrophages induces the polarization of M1 macrophages, secreting large amounts of inflammatory cytokines and exacerbating the inflammatory response, which contributes to necroptosis of tubular epithelial cells [88]. It could be presumed that macrophages regulate podocyte necroptosis in DN.

#### 4.2.5. Ferroptosis

Podocyte ferroptosis, a regulated type of cell death marked by iron-dependent lipid peroxidation, is essential in the pathogenesis of DN. In DN, dysregulated iron homeostasis causes iron overload in podocytes, promoting the formation of ROS and lipid radicals [89]. Moreover, hyperglycemia reduces glutathione levels and inhibits the activity of glutathione peroxidase 4, a key enzyme that prevents against lipid peroxidation, exacerbating lipid peroxidation in podocytes [90]. Podocyte ferroptosis facilitates podocyte loss, GFB damage, inflammation, and oxidative stress, all of which are critical factors in the progression of DN. Recent findings suggest that macrophages can transfer mitochondria to the neighboring cells and further mediate cell injury via triggering ferroptosis [91]. Considering the critical role of podocyte ferroptosis in DN, the effects of macrophages on podocyte ferroptosis under diabetic conditions needs to be further elucidated.

**Figure 2:**
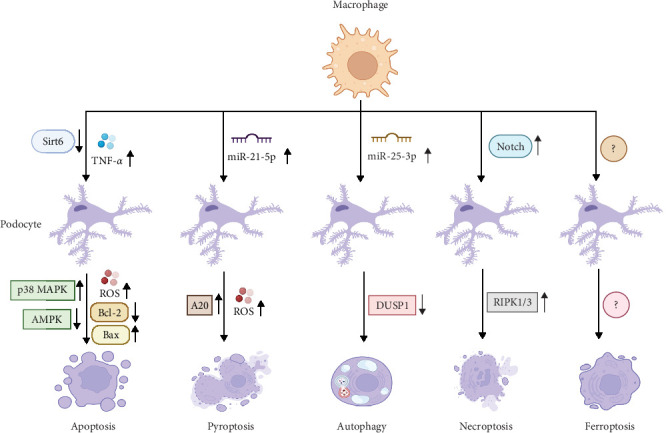
The effect of macrophages on death pathways of podocytes in DN. During DN progression, macrophages determine cell fate of podocytes through affecting multiple cell death processes, including apoptosis, pyroptosis, autophagy, necroptosis, and ferroptosis. AMPK, adenosine monophosphate-activated protein kinase; DUSP1, dual specificity protein phosphatase 1; MAPK, mitogen-activated protein kinase; NLRP3, the PYD domains-containing protein 3; RIPK1/3, receptor-interacting protein kinase 1 and 3; ROS, reactive oxygen species; TNF, tumor necrosis factor. ↑ indicates upregulation, ↓ indicates downregulation, and → indicates a promoting effect.

Altogether, macrophages determine cell fate of podocytes through affecting multiple cell death processes, including apoptosis, pyroptosis, autophagy, necroptosis, and ferroptosis (Figure 2). Hence, targeting macrophages to prevent against podocyte death may provide promising therapeutics for DN.

## 5. Regulation of miRNAs on the Cross Talk Between Macrophages and Podocytes in DN

**Figure 3:**
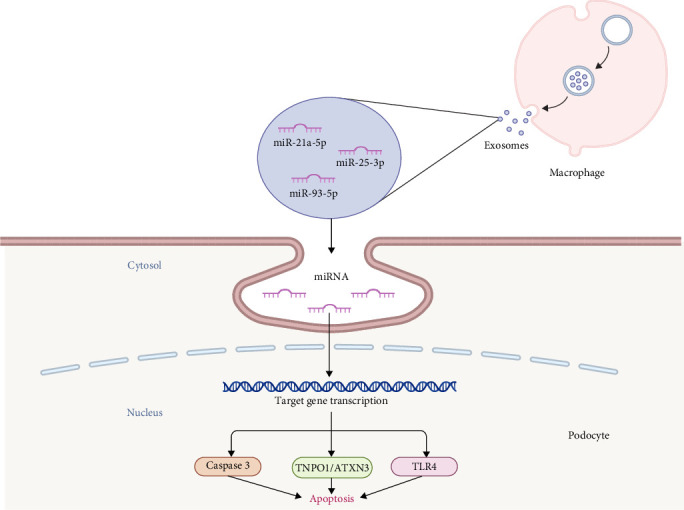
The regulatory role of macrophage-derived exosomes on podocytes DN. Macrophage-derived exosomes contain a variety of miRNAs, such as miR-21a-5p, miR-25-3p, and miR-93-5p, which enter into podocytes to target various genes and signaling pathways, influencing cell apoptosis. miRNA, microRNA; TLR4, Toll-like receptor 4; TNPO1/ATXN3, transportin-1/ataxin-3.

As concluded above, the interplay between macrophages and podocytes plays a significant role in the pathogenesis of DN. MiRNAs, small noncoding RNA molecules that modulate gene expression posttranscriptionally, have emerged as key regulators in this intercellular communication [92]. Recent studies have highlighted the transfer of miRNAs between macrophages and podocytes via extracellular vesicles. Macrophage-derived miRNAs can modulate gene expression in podocytes, influencing processes such as inflammation, fibrosis, and cell survival. For instance, the exosomes from macrophages treated with HG contain elevated levels of miR-21a-5p, which reduce the cell viability and promote the caspase-3-mediated apoptosis of podocytes, leading to glomerular structural damage, proteinuria, and renal dysfunction [93]. In addition, miRNAs derived from macrophages can regulate critical signaling pathways involved in podocyte survival. Zhuang et al. [94] unveiled that exosomes from HG-treated macrophages exacerbate HG-induced podocyte injury, as evidenced by reduced the proliferation capacity and enhanced apoptosis rate of podocytes; moreover, exosomal miR-21a-5p and miR-25-3p are verified to target the TNPO1/ATXN3 signal axis, which is responsible for the podocyte loss and disease progression. Analogously, TLR4 has been identified as a downstream target of miR-93–5p, which is enhanced in exosomes from M2 macrophages and mitigates LPS-induced podocyte apoptosis [95]. Thus, these results indicate that exosomal miRNAs derived from macrophages can affect podocyte survival (Figure 3).

In conclusion, targeting specific miRNAs offers a novel therapeutic approach for DN. miRNA mimics or inhibitors can be used to restore normal miRNA function or to inhibit the action of miRNAs implicated in disease pathogenesis.

## 6. Targeting Macrophages as Therapeutic Strategies for DN

Macrophages play a pivotal role in modulating podocyte injury and contribute to inflammation and fibrosis, which have been implicated in the pathogenesis of DN. Consequently, targeting macrophages to prevent against HG-induced podocyte loss presents a promising therapeutic strategy for DN (Table 1).

### 6.1. Modulating Macrophage Polarization

Therapies aimed at modulating macrophage polarization from a pro-inflammatory M1 phenotype to a reparative M2 phenotype can alleviate renal injury in DN. This approach involves targeting specific genes that govern macrophage polarization. For example, genetically modified macrophages stabilized by neutrophil gelatinase-associated lipocalin (NGAL) preserve their M2 phenotype, which elevates anti-inflammatory IL-10 and reduces renal TGF-*β*1 expression, as well as decreases infiltration of M1 macrophages, thus, mitigating podocyte loss and fibrosis [96]. Likewise, knockout of angiopoietin-like protein 3 drives the transformation of M1 type macrophages into M2 type macrophages, and further attenuates diabetes-related podocyte EMT and renal dysfunction [97]. However, diabetic kidneys with macrophage cyclooxygenase (COX)-2 deletion exhibits an increased M1 macrophage phenotype, which causes renal infiltration of pro-inflammatory cells, endoplasmic reticulum stress, podocytes loss, and fibrosis, developing severe DN [98]. Hence, modulation of macrophage polarization by gene editing can meliorate podocytes loss and DN progression.

Several drugs and natural agents have been shown to affect macrophage polarization and exert a protective role in DN. For instance, calcitriol, a bioactive 1,25-dihydroxyvitamin D3, converts HG-mediated M1 macrophages toward an M2 phenotype in DN rats, thereby reversing podocyte injury [99]. *Trichosanthes kirilowii* lectin, an herb that exhibits antidiabetic activities, is verified to abate deterioration in renal structure and function of DN rats by increasing the proportion of M2 macrophage through suppression of the Notch signaling [100]. Similarly, thalidomide, a drug for the treatment of plasma cell myeloma, can promote M2 macrophage differentiation by reducing the TNF-*α* and IL-1*β* levels, thereby hampering HG-induced podocyte injury [101]. Besides, hyperoside, a traditional Chinese herb with antioxidative, antiapoptotic properties, and podocyte-protective effects, can modulate macrophage polarization by converting pro-inflammatory M1 macrophages into anti-inflammatory M2 ones, repressing renal inflammatory response and production of pro-inflammatory cytokines, such as MCP-1 and TNF-*α* [102]. Therefore, promoting M2 macrophage transformation via herbs or drugs is effective to protect against podocyte injury.

### 6.2. Inhibiting the Pro-Inflammatory Activity of Macrophages

In DN, hyperglycemia and the diabetic milieu promote the recruitment and activation of M1 macrophages within the kidney. These activated macrophages secrete pro-inflammatory cytokines, chemokines, and growth factors, exacerbating renal injury. Strategies aimed at inhibiting macrophage recruitment and activation to the kidney have shown promise in mitigating disease progression. To investigate the function of mucin-domain containing-3 (Tim-3) on macrophage activation, Yang et al. [103] revealed that the expression of Tim-3 is increased on renal macrophages in patients with DN, which is associated with renal dysfunction; moreover, upregulated Tim-3 in macrophages expedites podocyte injury via activating the NF-*κ*B/TNF-*α* signaling pathway. Suppression of macrophage activation through knockdown of Tim-3 alleviates renal damage in DN mice [103]. Coincidentally, administration of DN mice with ISO-1, an inhibitor of macrophage migration inhibitory factor, restrains the activation of macrophages and production of pro-inflammatory cytokines, thus, decreasing podocyte damage and renal fibrosis [104]. Additionally, treatment of sulfated cholecystokinin octapeptide, which exerts renoprotective effects through its anti-inflammatory actions, impedes infiltration of macrophages and expression of pro-inflammatory genes, which reduce podocyte loss and albuminuria in diabetic rats [105]. These findings imply that repression of the activation of pro-inflammatory macrophages decreases podocyte damage and improves renal function.

## 7. Conclusion and Perspective

As essential components of GFB, podocytes play a pivotal role in the glomerular filtration function and podocyte loss is a key pathological feature in DN. Macrophages are regarded as a kind of versatile innate immune cells, exerting crucial functions in kidney homeostasis and DN progression. The heterogeneity of phenotypes and functions on renal macrophages affect the pathogenesis of DN. A critical aspect of these effects is the cross talk between macrophages and podocytes, wherein macrophages affect podocyte survival by regulating podocyte apoptosis, pyroptosis, autophagy, necroptosis, and ferroptosis. Advances in the research of macrophage-derived miRNAs have revealed complex regulatory networks that govern gene expression and secretion of cytokines and chemokines in podocytes and offers insights into the pathophysiology and potential therapeutics of DN. Hence, targeting macrophages to promote podocyte survival, through modulation of macrophage polarization and inhibition of pro-inflammatory macrophage activation, has emerged as a novel therapeutic strategy. However, the interplay between macrophages and podocytes is highly complex and molecular mechanisms are not fully understood. For example, whether macrophages directly regulate podocyte necroptosis and ferroptosis is still elusive. Besides, effective macrophage-targeted therapies in DN must be specific and safe, minimizing off-target effects while preserving the beneficial roles of macrophages in renal inflammation and tissue repair. Given the multifaceted role of macrophages in regulating podocyte survival, combination therapies that target multiple aspects of DN, including podocyte protection, inflammation reduction, and fibrosis prevention, may offer enhanced therapeutic efficacy. In addition, utilizing genomics, proteomics, and metabolomics to study the cross talk between macrophages and podocytes can provide a more comprehensive understanding of DN pathogenesis.

## Figures and Tables

**Table 1:** Interventions for modulating macrophage polarization and activation in DN.

Intervention	Target in macrophages	Related signaling pathways or cytokines	Outcome	Ref.
Gene modification	NGAL↑	IL-10↑; TNF-*α*, IL-1*β*, TGF-*β*1↓	Reducing podocyte loss, albuminuria, and renal fibrosis	[96]
Gene modification	Angptl3↓	NLRP3, IL-1*β*↓	Attenuating podocyte EMT and renal dysfunction	[97]
Gene modification	COX-2↓	iNOS, NF-*κ*B↑; IL-4R*α*↓	Promoting endoplasmic reticulum stress and of podocyte loss	[98]
Calcitriol	Unknown	iNOS, TNF-*α*↓	Ameliorating podocyte injury, proteinuria, and renal damage	[99]
TKL	Notch↓	L-10↑; iNOS, TNF-*α*↓	Increasing proliferation potential of podocytes and restoring renal function and structure	[100]
Thalidomide	Unknown	TNF-*α*, IL-1*β*↓	Reducing HG-induced podocyte injury	[101]
Hyperoside	Unknown	Arg-1↑; MCP-1, iNOS, TNF-*α*↓	Lessening albuminuria and glomerular mesangial matrix expansion	[102]
Gene modification	Tim-3↓	NF-*κ*B/TNF-*α*↓	Ameliorating diabetic renal injury	[103]
ISO-1	MIF↓	TNF-*α*, IL-1*β*, IL-6↓	Blocking podocyte damage and albuminuria	[104]
Gene modification	CCKR↓	TNF-*α*↓	Mitigating podocyte loss, albuminuria, and inflammation	[105]

*Note*: ↑ indicates upregulation and ↓ indicates downregulation.

Abbreviations: Angptl3, angiopoietin-like protein 3; Arg, arginase; CCKR, cholecystokinin receptor; COX, cyclooxygenase; EMT, epithelial-to-mesenchymal transition; HG, high glucose; IL, interleukin; IL-4R*α*, interleukin-4 receptor-*α*; iNOS, inducible nitric oxide synthase; ISO-1, MIF inhibitor; MCP-1, monocyte chemotactic protein-1; MIF, migration inhibitory factor; NF, nuclear factor; NGAL, neutrophil gelatinase-associated lipocalin; NLRP3, NOD-like receptor family pyrin domain containing 3; Tim-3, T cell immunoglobulin and mucin domain-containing protein 3; TKL, Trichosanthes kirilowii lectin; TNF, tumor necrosis factor.

## Data Availability

The data that support the findings of this study are available from the corresponding author upon reasonable request.

## Nomenclature

AGEs: Advanced glycation end products
ATGs: Autophagy-related genes
COX: Cyclooxygenase
EMT: Epithelial-to-mesenchymal transition
GSDMD: Gasdermin D
GH: Growth hormone
iNOS: Inducible nitric oxide synthase
IL: Interleukin
MCP-1: Monocyte chemotactic protein-1
PKC: Protein kinase C
ROS: Reactive oxygen species
SD: Slit diaphragm
TGF: Transforming growth factor
VEGF: Vascular endothelial growth factor
Arg: Arginase
CXCL: CXC chemokine ligand
DN: Diabetic nephropathy
FPs: Foot processes
GFB: Glomerular filtration barrier
HG: High glucose
IFN: Interferon
miRNAs: MicroRNAs
NF: Nuclear factor
NLRP3: PYD domains-containing protein 3
RIPK: Receptor-interacting protein kinase
TLRs: Toll-like receptors
TNF: Tumor necrosis factor.
